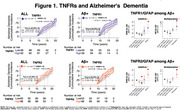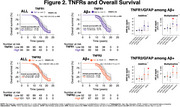# Synergistic effects of GFAP with TNFR1 and TNFR2 on conversion to AD dementia and overall survival across the Alzheimer’s disease spectrum

**DOI:** 10.1002/alz70862_110252

**Published:** 2025-12-23

**Authors:** Laura Alejandra Ramirez Tirado, Ann D Cohen, C. Elizabeth Shaaban, Cristy Matan, Brian J Lopresti, Milos D. Ikonomovic, Thomas K Karikari, Victor L Villemagne, Oscar L Lopez, Oscar L Lopez

**Affiliations:** ^1^ University of Pittsburgh, Pittsburgh, PA USA; ^2^ University of Pittsburgh Alzheimer's Disease Research Center (ADRC), Pittsburgh, PA USA; ^3^ University of Pittsburgh School of Medicine, Pittsburgh, PA USA; ^4^ VA Pittsburgh Healthcare System, Pittsburgh, PA USA; ^5^ UPMC, Pittsburgh, PA USA; ^6^ Cognitive Disorders & Comprehensive Alzheimer's Disease Center, Thomas Jefferson University, Philadelphia, PA USA

## Abstract

**Background:**

We recently reported synergistic effects of TNFR1 and TNFR2 with astrogliosis resulting in greater vascular burden and neurodegeneration, particularly in Aβ+ participants. Here, we aimed to assess the interactions of peripheral inflammation and astrogliosis on incidence of AD dementia and mortality. We hypothesized synergistic effects with astrogliosis resulting in increased incidence of dementia, and mortality, particularly in Aβ+ participants.

**Methods:**

Participants of the Gingko evaluation of memory (GEM) study underwent PiB‐PET scans between 2009‐2018. GFAP and peripheral inflammatory markers were measured in 2009 by immunoassay. We evaluated the relationship of peripheral markers of inflammation (TNFR1,TNFR2) and astrogliosis (GFAP</≥196pg/mL) on AD dementia incidence and mortality using Cox proportional hazards models adjusted for age, sex, education, *APOEε4*, cystatin C and baseline Aβ status.

**Results:**

We included 190 participants (mean age:86±2.8 yrs., 40.8% women, 96.9% White); 53% developed AD dementia over a median time of 15.78 years (95% CI:12.16–19.26). After full adjustment, Aβ+ status was associated with greater hazards for dementia HR:1.73(95%CI:1.10–2.70); *p* =.016. High levels of TNFR1 HR:1.41(95%CI:0.88–2.27); *p* =.145 or TNFR2 HR:1.04(95%CI:0.65–1.65); *p* =.855 were not associated with higher hazards for dementia after full adjustment. Likewise, among Aβ+ participants, high levels of TNFR1 and TNFR2 were also not associated with greater hazards for AD dementia. However, among Aβ+ participants, fully adjusted models showed greater hazards of dementia and significant additive and multiplicative interactions for the joint effects of GFAP with TNFR1 HR:4.55(95%CI:1.14–18.19); *p* =.032 and with TNFR2 HR:4.07(95%CI:1.35–12.24); *p* =.012. Regarding mortality, median survival was 16 years(95%CI:13.39‐18.58). We found no difference in mortality by amyloid status HR:1.18(95%CI:0.79–1.75); *p* =.405, GFAP or NfL status. High levels of TNFR1 HR:1.50(95%CI:1.00‐2.24); *p* =.046, but not TNFR2 HR:1.20(95%CI:0.80–1.80); *p* =.365, were associated with greater hazards of death after adjustment. Among Aβ+ participants high levels of TNFR1 were also associated with worse survival. Fully adjusted models also showed greater hazards of death for the joint effects of GFAP with TNFR1 and TNFR2, and a synergistic effect on the additive scale was found between GFAP and TNFR2 (Excess relative risk due to interaction:1.43;95%CI:‐.09–2.76; *p* =0.036).

**Conclusion:**

Interaction of GFAP with TNFR1 and TNFR2 seems to be clinically relevant for progression to AD dementia and mortality in Aβ+ participants.